# Supplementary material for: Lysogen formation governs colonies while lytic infection is more prevalent in single cells of the bloom-forming cyanobacterium, Microcystis
Source: bioRxiv. 2025 Feb 13:2025.02.12.637950. Preprint. [Version 1] doi: 10.1101/2025.02.12.637950 (PMC11844469; doi:10.1101/2025.02.12.637950)
Supplement: Supplement 1 [file media-1.pdf]

Supplementary Information for:

Lysogen formation governs colonies while lytic infection is more prevalent in single cells of the bloom-forming cyanobacterium, *Microcystis*

X. Huang[1,2], EE Chase[2], BN Zepernick[2], RM Martin[2], LE Krausfeldt[2], HL Pound[2], H. Wu[1], Z. Zheng[*1] and SW Wilhelm[*2]

1. Department of Environmental Science and Engineering, Fudan University, Shanghai, PR China
2. Department of Microbiology, The University of Tennessee, Knoxville TN

Address for contact information: wilhelm@utk.edu, zzhenghj@fudan.edu.cn

METHODS

Collection of 2018 samples

Surface water samples were collected from a dock that stretches approximately 100 yards into Meilang Bay near the TLLER field station on *Taihu* on 08/18/2018 at 13:00 and 08/19/2018 at 7:00. To enrich for colonies, water was concentrated on a 28 µm mesh while the < 28 µm filtrate was captured as the representative single-cell community. Duplicate samples were collected at each time point, and both fractions were filtered through 0.2 µm Sterivex, preserved with RNA*later* and stored at -80° C until processing.

RNA extraction and sequencing of 2018 samples

Total RNA was extracted using a previously described phenol–chloroform method followed by ethanol precipitation[1]. Genomic DNA was removed using the Turbo DNA-free kit (Ambion, Austin, TX, USA). RNA integrity and concentration were assessed with the Qubit RNA HS Assay Kit (Invitrogen, Waltham, MA, USA). Samples exhibiting residual DNA, as indicated by Qubit quantification, underwent an additional DNase treatment. Purified RNA was subsequently subjected to library preparation, rRNA depletion, and sequencing on the Illumina NextSeq platform using the Epidemiology Ribo-Zero Gold rRNA Removal Kit.

Sampling site	Date	Time	Longitude	Latitude	Colonial samples	Single-cell samples
S1	8/26/2023	10:02	120°01’21’’E	31°24’48’’N	C_S1_1<br>C_S1_2<br>C_S1_3	S_S1_1<br>S_S1_2<br>S_S1_3
S2	8/26/2023	10:13	120°00’10’’E	31°23’28’’N	C_S2_1<br>C_S2_2<br>C_S2_3	S_S2_1<br>S_S2_2<br>S_S2_3
S3	8/26/2023	10:27	119°58’30’’E	31°20’55’’N	C_S3_1<br>C_S3_2<br>C_S3_3	S_S3_1<br>S_S3_2<br>S_S3_3
S4	8/26/2023	11:15	120°02’43’’E	31°23’51’’N	C_S4_1<br>C_S4_2<br>C_S4_3	S_S4_1<br>S_S4_2<br>S_S4_3

Supplementary Table 1: Information of each sampling site. The first letter of each sample name indicates the morphology of the sample, with C representing colony and S representing single cell. The second segment represents the sampling site (e.g., S2), and the last number indicates the replicate number.

Organism Scientific Name	Organism Qualifier	Taxonomy id	Assembly Accession	Source	Size	Gene Count
Microcystis aeruginosa NIES-843	strain: NIES-843	449447	GCF_000010625.1	RefSeq	5842795	5792
Microcystis aeruginosa NIES-2549	strain: NIES-2549	1641812	GCF_000981785.2	RefSeq	4301200	4087
Microcystis panniformis FACHB-1757	strain: FACHB-1757	1638788	GCF_001264245.1	RefSeq	5686839	5640
Microcystis aeruginosa NIES-2481	strain: NIES-2481	1698524	GCF_001704955.2	RefSeq	4440545	4242
Microcystis aeruginosa PCC 7806SL	strain: PCC 7806SL	1903187	GCF_002095975.1	RefSeq	5139339	4936
Microcystis sp. MC19	strain: MC19	1967666	GCF_003019735.1	RefSeq	5020243	4934
Microcystis viridis NIES-102	strain: NIES-102	213615	GCF_003945305.1	RefSeq	5874197	5786
Microcystis aeruginosa FD4	strain: FD4	2686288	GCF_009792235.1	RefSeq	5493112	5530
Microcystis aeruginosa NIES-298	strain: NIES-298	449468	GCF_010196425.1	RefSeq	5015081	4823
Microcystis aeruginosa	strain: NIES-88	1126	GCF_019704275.1	RefSeq	5501105	5588
Microcystis aeruginosa FACHB-905 = DIANCHI905	strain: DIANCHI905	267865	GCF_021172085.1	RefSeq	5103104	4909
Microcystis aeruginosa str. Chao 1910	strain: Chao 1910	2945101	GCF_026222535.1	RefSeq	5669822	5720
Microcystis aeruginosa PCC 7806	strain: PCC 7806	267872	GCF_030553035.1	RefSeq	5103923	4908
Microcystis aeruginosa NRERC-214	strain: NRERC-214	2528657	GCF_031754835.1	RefSeq	4981678	4671
Microcystis aeruginosa 1339	strain: 1339	3113711	GCF_038396575.1	RefSeq	4749293	4559
Microcystis aeruginosa PCC 7806	strain: PCC 7806	267872	GCF_041506625.1	RefSeq	5096229	4894

Supplementary Table 2: Summary of 16 complete *Microcystis* genomes used for the construction of the *Microcystis* pangenome.

Sample	Raw reads count	Total bases of raw reads	Trimmed reads count	Trimmed reads percentage (%)	mRNA reads count	mRNA reads percentage (%)	Reads mapped to the *Microcystis* pangenome count	*Microcystis* pangenome -mapped reads percentage (%)	Reads mapped to the co-assembly count	Co-assembly-mapped reads percentage (%)
C_S1_1	1.55E+08	2.33E+10	1.52E+08	98.17%	1.52E+08	99.93%	5.53E+07	36.49%	1.34E+08	88.29%
C_S1_2	1.58E+08	2.39E+10	1.54E+08	97.50%	1.54E+08	99.92%	6.85E+07	44.43%	1.4E+08	90.65%
C_S1_3	9.74E+07	1.47E+10	9.53E+07	97.86%	9.52E+07	99.95%	3.32E+07	34.9%	8.39E+07	88.11%
C_S2_1	1.4E+08	2.12E+10	1.37E+08	97.95%	1.37E+08	99.75%	5.13E+07	37.41%	1.23E+08	90.03%
C_S2_2	1.18E+08	1.78E+10	1.15E+08	97.57%	1.15E+08	99.71%	4.37E+07	38.1%	1.03E+08	89.79%
C_S2_3	8.99E+07	1.36E+10	8.78E+07	97.69%	8.78E+07	99.96%	2.79E+07	31.79%	7.83E+07	89.16%
C_S3_1	1.4E+08	2.11E+10	1.32E+08	94.90%	1.31E+08	99.05%	5.95E+07	45.38%	1.21E+08	92.48%
C_S3_2	1.67E+08	2.52E+10	1.63E+08	97.77%	1.63E+08	99.84%	7.31E+07	44.89%	1.5E+08	91.97%
C_S3_3	9.68E+07	1.46E+10	9.58E+07	98.98%	9.43E+07	98.35%	4.3E+07	45.6%	8.61E+07	91.31%
C_S4_1	1.35E+08	2.04E+10	1.28E+08	94.87%	1.28E+08	99.94%	5.61E+07	43.8%	1.17E+08	91.09%
C_S4_2	1.08E+08	1.62E+10	1.06E+08	98.37%	1.06E+08	99.90%	4.17E+07	39.45%	9.66E+07	91.36%
C_S4_3	9.6E+07	1.45E+10	9.36E+07	97.44%	9.35E+07	99.95%	3.42E+07	36.54%	8.3E+07	88.75%
S_S1_1	1.15E+08	1.74E+10	1.12E+08	97.78%	1.12E+08	99.94%	3.59E+07	31.95%	9.83E+07	87.52%
S_S1_2	1.14E+08	1.73E+10	1.11E+08	97.19%	1.11E+08	99.92%	2.21E+07	19.93%	8.98E+07	80.82%
S_S1_3	1.14E+08	1.72E+10	1.1E+08	96.68%	1.1E+08	99.89%	2.96E+07	26.86%	9.32E+07	84.52%
S_S2_1	1.13E+08	1.7E+10	1.1E+08	97.07%	1.09E+08	99.87%	1.62E+07	14.84%	8.46E+07	77.29%
S_S2_2	1.08E+08	1.63E+10	1.06E+08	98.21%	1.06E+08	99.93%	1.27E+07	11.99%	8.49E+07	80.36%
S_S2_3	1.02E+08	1.54E+10	9.99E+07	98.08%	9.97E+07	99.84%	1.72E+07	17.25%	8.53E+07	85.53%
S_S3_1	1.37E+08	2.07E+10	1.34E+08	97.43%	1.34E+08	99.95%	2.7E+07	20.19%	1.18E+08	88.27%
S_S3_2	1.46E+08	2.21E+10	1.43E+08	97.79%	1.43E+08	99.93%	3.35E+07	23.41%	1.28E+08	89.4%
S_S3_3	1.36E+08	2.06E+10	1.33E+08	97.52%	1.33E+08	99.93%	3.5E+07	26.36%	1.19E+08	89.36%
S_S4_1	1.26E+08	1.9E+10	1.22E+08	96.46%	1.21E+08	99.87%	1.48E+07	12.19%	9.7E+07	79.93%
S_S4_2	1.38E+08	2.08E+10	1.33E+08	96.26%	1.32E+08	99.83%	1.73E+07	13.03%	1.08E+08	81.65%
S_S4_3	1.02E+08	1.54E+10	9.95E+07	97.68%	9.94E+07	99.91%	1.07E+07	10.73%	8.02E+07	80.61%

Supplementary Table 3: Summary of sequence information for Lake Taihu metatranscriptomic libraries collected on August 26, 2023. The first letter of each sample name indicates the morphology of the sample, with C representing colony and S representing single cell. The second segment represents the sampling site (e.g., S2), and the last number indicates the replicate number.

Supplementary Figure 1

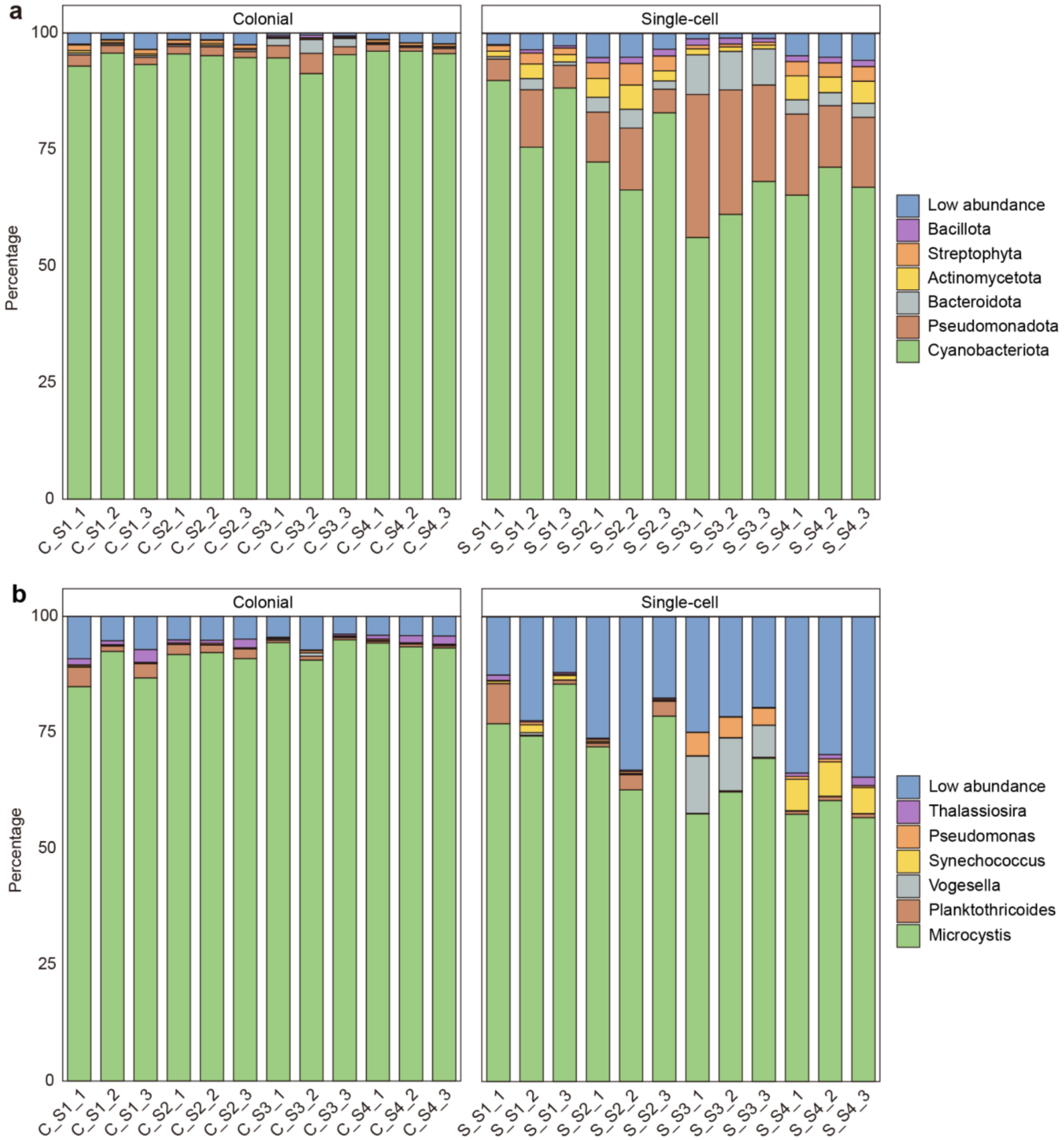

Supplementary Figure 1: Phylum- (**a**) and genus-level (**b**) distribution of colonial and single-cell samples in the 2023 database. Each bar represents a single sample, with the relative abundance of taxa shown as a percentage. The number of samples is as follows: colonial (n = 12) and single-cell (n = 12).

Supplementary Figure 2

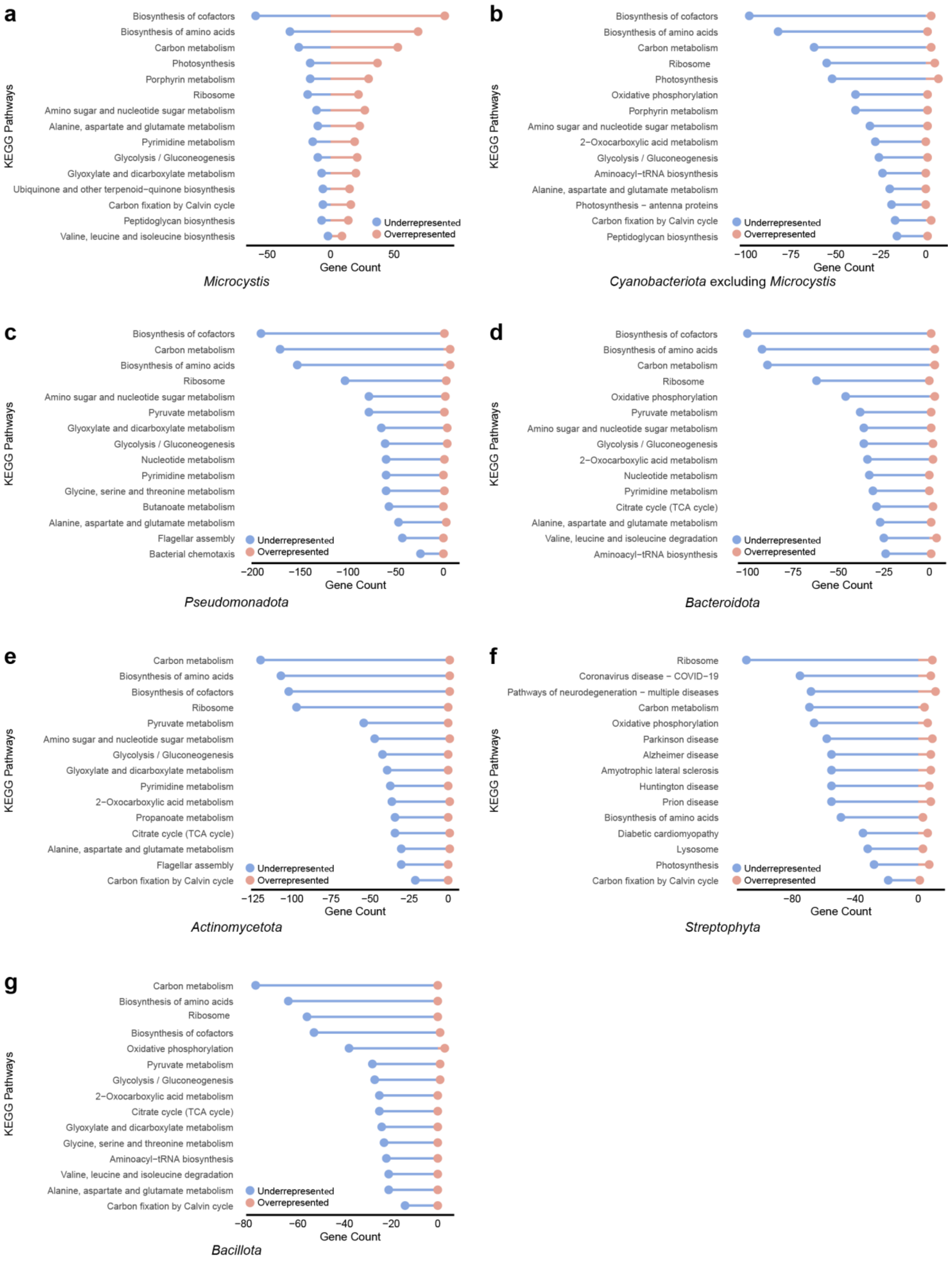

Supplementary Figure 2: Top 15 KEGG pathways enriched in the top six phyla based on differential gene expression analysis. The phyla include **a** *Microcystis*, **b** *Cyanobacteriota* (excluding *Microcystis*), **c** *Pseudomonadota*, **d** *Bacteroidota*, **e** *Actinomycetota*, and **g** *Bacillota*. Each panel shows the number of overrepresented (red) and underrepresented (blue) genes in key metabolic and functional pathways. KEGG pathways are ranked by gene count. *Cyanobacteriota* was divided into *Microcystis* and non-*Microcystis* *Cyanobacteriota*.

Supplementary Figure 3

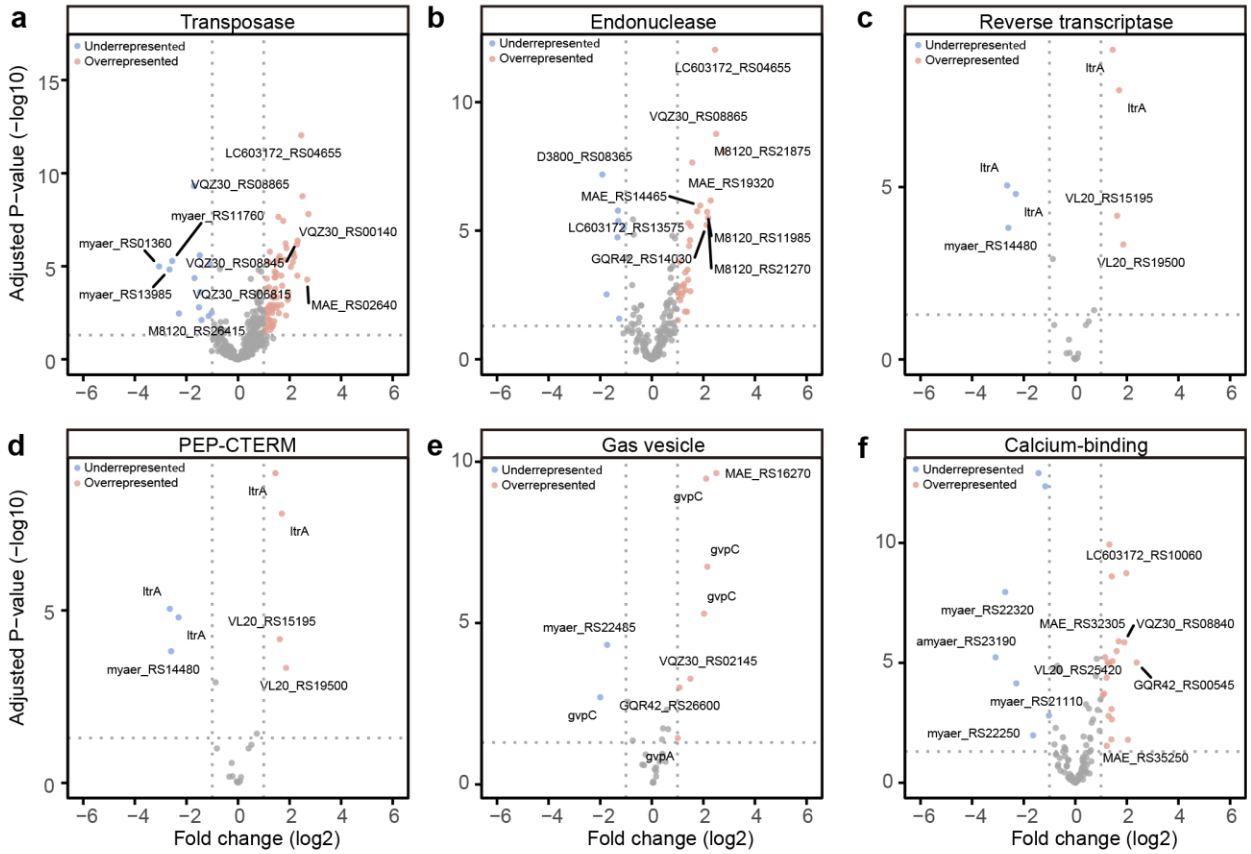

Supplementary Figure 3: Volcano plots depicting the differential expression and significance of genes across colonial and single-cell samples. **a**, **b**, and **c** highlight key genes categorized under COG category L, specifically transposase (**a**), endonuclease (**b**), and reverse transcriptase (**c**). **d**, **e**, and **f** present genes without COG classification, showcasing PEP-CTERM (**d**), gas vesicle proteins (**e**), and calcium-binding proteins (**f**). Red and blue points denote overrepresented and underrepresented genes, respectively, while gray points represent non-significant genes (adjusted p-value > 0.05). Annotated points indicate specific genes of interest.

1. Zepernick, B. N. *et al.* Declines in ice cover are accompanied by light limitation responses and community change in freshwater diatoms. *ISME J.* **18**, wrad015 (2024).
